# On the expansion of “dangerous” gene families in vertebrates

**DOI:** 10.1186/1471-2105-15-S3-A4

**Published:** 2014-02-11

**Authors:** Séverine Affeldt, Param Priya Singh, Giulia Malaguti, Hervé Isambert

**Affiliations:** 1 Institut Curie, Research Center, CNRS UMR168 UPMC, 26, rue d’Ulm, 75005 Paris, France

## Background

“Dangerous” gene families, defined as prone to dominant (gain-of-function) mutations, have been greatly expanded in the course of vertebrate evolution by contrast to gene families more prone to recessive (loss-of-function) mutations. While the maintenance of “essential” genes is ensured by their lethal double null mutations, the expansion of “dangerous” gene families, implicated in cancer and other severe genetic diseases in human, remains puzzling. Could gene susceptibility to dominant deleterious mutations be somehow responsible for this striking evolutionary expansion of “dangerous” gene families?

## Results

We proposed such an evolutionary model suggesting that this counterintuitive expansion of “dangerous” gene families is in fact a consequence of their susceptibility to deleterious mutations and purifying selection in polyploid species that arose from two rounds of whole genome duplication (WGD) events dating back from the onset of jawed vertebrates, some 500MY ago [1,2]. All WGD duplicates, so-called "ohnologs", were thus initially acquired by speciation without the need to provide evolutionary benefit to be fixed in post-WGD species.

Our data mining analyses, based on the 20,506 human protein coding genes, first revealed a strong correlation between the retention of ohnologs and their susceptibility to dominant deleterious mutations in humans [3]. It appears that the human genes associated with the occurrence of cancer and other genetic diseases (8,095) have retained significantly more ohnologs than expected by chance (48% *versus* 35%; 48% : 3,844/8,095; P=1.3×10^−128^, χ^2^). We also found that the retention of ohnologs is more strongly related to their “dangerousness” than their “essentiality” [3].

To go beyond mere correlations, we also performed mediation analyses, following the approach of Pearl [4], and quantified the *direct* and *indirect* effects of many genomic properties, such as essentiality, expression levels or divergence rates, on the retention of ohnologs.

**Figure 1:**
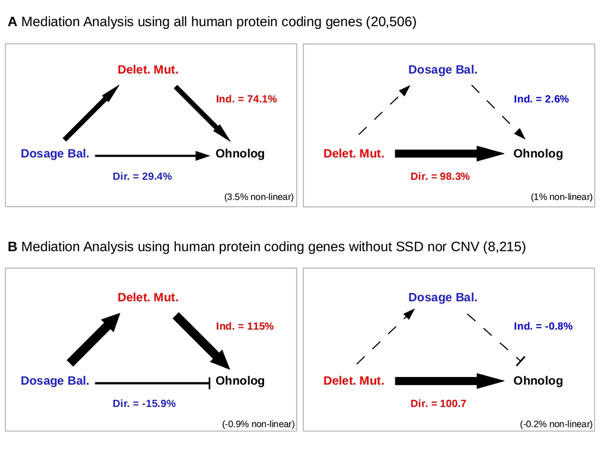
Quantitative Mediation analysis of direct versus indirect effects of deleterious mutations and dosage balance on the retention of human ohnologs using (A) all human protein coding genes (20,506) or (B) human protein coding genes without SSD nor CNV (8,215). The thickness of the arrows outlines the relative importance of the corresponding direct or indirect effects. Dir.< 0 or Ind.<0 corresponds to an anticorrelated direct or indirect effect, respectively. Gene prone to deleterious mutations and/or to dosage balance (including haploinsufficient genes and genes involved in multiprotein complexes) are taken from [3].

This enabled us to investigate an alternative hypothesis frequently invoked to account for the biased retention of ohnologs, namely the “dosage-balance” hypothesis [5]. While this hypothesis posits that the ohnologs are retained because their interactions with protein partners require to maintain balanced expression levels throughout evolution, we found that most of the ohnologs have in fact been eliminated from permanent complexes in human (7.5% *versus* 35%; 7.5% : 18/239; P=1.2×10^−18^, χ^2^). These mediation analyses also showed (Fig. 1) that the gene susceptibility to deleterious mutations is more relevant than dosage-balance for the retention of ohnologs in more transient complexes.

## Conclusions

These results suggest that the retention of human ohnologs is primarily caused by their susceptibility to deleterious mutations. They further establish that the retention of many ohnologs suspected to be dosage balanced is in fact *indirectly mediated* by their susceptibility to dominant deleterious mutations.

All in all, this supports a new evolutionary model relying on a non-adaptive mechanism that hinges on (*i*) the *speciation* event concomitant to WGD, and (*ii*) the *dominance* of deleterious mutations leading to purifying selection in post-WGD species.
